# Supplementary material for: Smooth Interpolating Curves with Local Control and Monotone Alternating Curvature
Source: Comput Graph Forum. 2022 Oct 6;41(5):25–38. doi: 10.1111/cgf.14600 (PMC9827861; doi:10.1111/cgf.14600)
Supplement: Supplementary file 1 — Supplement Material [file CGF-41-25-s001.zip › Local-Smooth-Interpolating-MonoCurvature/extern/clothoids/submodules/Utils/ThirdParties/fmt/fmt-8.0.1.zip!/fmt-8.0.1/doc/_templates/layout.html]

{% extends "!layout.html" %}
{% block extrahead %}


{# Google Analytics #}
{% endblock %}
{%- macro searchform(classes, button) %}

{% if button %}
{% endif %}
{%- endmacro %}
{% block header %}

{# Brand and toggle get grouped for better mobile display #}

Toggle navigation

{fmt}

{# Collect the nav links, forms, and other content for toggling #}

- {{ version }}
  {% for v in versions.split(',') %}- {{v}}
  {% endfor %}
{% for name in ['Contents', 'Usage', 'API', 'Syntax'] %}
{% if pagename == name.lower() %}- {{name}}
  (current)
{%else%}- {{name}}
{%endif%}
{% endfor %}
{% if pagename != 'search' %}
{{ searchform('navbar-form navbar-right', False) }}
{%endif%}

{# /.navbar-collapse #}

{# /.col-md-offset-2 #}

{# /.row #}

{# /.tb-container #}
{% if pagename == "index" %}
{% set download\_url = 'https://github.com/fmtlib/fmt/releases/download' %}

# {fmt}

A modern formatting library

{% set name = 'fmt' if version.split('.')[0]|int >= 3 else 'cppformat' %}
 Download


{% for v in versions.split(',') %}
{% set name = 'fmt' if v.split('.')[0]|int >= 3 else 'cppformat' %}- Version {{v}}
{% endfor %}

{% endif %}
{% endblock %}
{# Disable relbars. #}
{% block relbar1 %}
{% endblock %}
{% block relbar2 %}
{% endblock %}
{% block content %}

{# Sidebar is currently disabled.

{%- block sidebarlogo %}
{%- if logo %}

{%- endif %}
{%- endblock %}
{%- for sidebartemplate in sidebars %}
{%- include sidebartemplate %}
{%- endfor %}

#}

{% block body %} {% endblock %}

{% endblock %}
{% block footer %}
{{ super() }}
{# Placed at the end of the document so the pages load faster. #}
{% endblock %}
